# Glia Modulates Immune Responses in the Retina Through Distinct MHC Pathways

**DOI:** 10.1002/glia.24656

**Published:** 2025-01-28

**Authors:** Simona Intonti, Despina Kokona, Martin S. Zinkernagel, Volker Enzmann, Jens V. Stein, Federica M. Conedera

**Affiliations:** ^1^ Department of Experimental and Clinical Medicine University of Florence Florence Italy; ^2^ Department of Ophthalmology, Bern University Hospital and Department of BioMedical Research University of Bern Bern Switzerland; ^3^ Department of Oncology, Microbiology and Immunology University of Fribourg Fribourg Switzerland

**Keywords:** antigen‐presenting cells, microglia, Müller cells, retina, T‐cells

## Abstract

Glia antigen‐presenting cells (APCs) are pivotal regulators of immune surveillance within the retina, maintaining tissue homeostasis and promptly responding to insults. However, the intricate mechanisms underlying their local coordination and activation remain unclear. Our study integrates an animal model of retinal injury, retrospective analysis of human retinas, and in vitro experiments to gain insights into the crucial role of antigen presentation in neuroimmunology during retinal degeneration (RD), uncovering the involvement of various glial cells, notably Müller glia and microglia. Glial cells act as sentinels, detecting antigens released during degeneration and interacting with T‐cells via MHC molecules, which are essential for immune responses. Microglia function as APCs via the MHC Class II pathway, upregulating key molecules such as Csf1r and cytokines. In contrast, Müller cells act through the MHC Class I pathway, exhibiting upregulated antigen processing genes and promoting a CD8^+^ T‐cell response. Distinct cytokine signaling pathways, including TNF‐α and IFN Type I, contribute to the immune balance. Human retinal specimens corroborate these findings, demonstrating glial activation and MHC expression correlating with degenerative changes. In vitro assays also confirmed differential T‐cell migration responses to activated microglia and Müller cells, highlighting their role in shaping the immune milieu within the retina. In summary, our study emphasizes the involvement of retinal glial cells in modulating the immune response after insults to the retinal parenchyma. Unraveling the intricacies of glia‐mediated antigen presentation in RD is essential for developing precise therapeutic interventions for retinal pathologies.

## Main Points

- Glial cells as antigen‐presenting cells are crucial for retinal immune responses during degeneration.
- Microglia and Müller cells use distinct MHC pathways to modulate T‐cell responses.
- Several cytokine signaling pathways maintain immune balance.

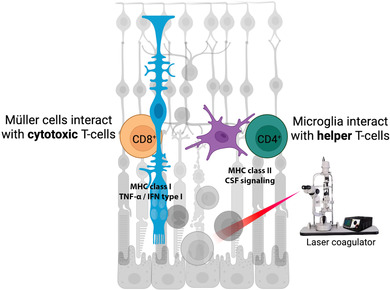

## 1 Introduction

The eye was once thought to be an immune privileged site; however, this notion is increasingly challenged by evidence of T‐cell infiltration in many retinal diseases (Camelo 2014). This prompts further questions into the role of the immune system in the development of retinal diseases. While the role of T‐cells in diseases such as uveitis is well established, their involvement in retinal degeneration (e.g., age‐related macular degeneration [AMD]) remains poorly explored to date. We provided significant evidence of T‐cell accumulation in ocular tissues of humans and demonstrated the harmful effects of cytotoxic (CD8^+^) T‐cells on retinal degeneration (RD) in a murine model of focal injury (Conedera et al. 2023). While these data suggest T‐cells are implicated in RD, the precise mechanisms of their activation and interaction remain elusive, indicating a need to explore how antigen‐presenting cells (APCs) orchestrate adaptive immune responses within the retina.

To enable the immune system to recognize and respond to insults, the major histocompatibility complex (MHC) plays a critical role in antigen presentation. The MHC is divided into two classes: MHC Class I and MHC Class II. MHC Class I molecules are expressed on all nucleated cells and primarily present intracellular (intrinsic) antigens to CD8+ cytotoxic T cells. This allows for immune surveillance of virtually all body tissues. In contrast, MHC Class II molecules are expressed on specialized cells known as APCs, which include B cells, dendritic cells, and macrophages/microglia. These APCs process and present extracellular (extrinsic) antigens via MHC Class II to CD4+ helper T cells (Liu et al. 2024; Wieczorek et al. 2017).

The retina harbors two main subsets of glia known as microglia and macroglia. Both glia cell types play pivotal roles in immune responses by actively surveying their microenvironment, presenting antigens to T‐cells, and orchestrating immune tolerance (Reichenbach and Bringmann 2020). Under physiological conditions, microglia exhibit a ramified morphology and continuously scan the retinal parenchyma using motile protrusions, playing a vital role in immune surveillance (Verkhratsky, Sun, and Tanaka 2021). This enables microglia to promptly detect abnormalities within retinal tissue, facilitating rapid responses to potential threats. During degeneration, microglia become activated in response to stimuli such as inflammation or tissue damage. Consequently, they release factors and express receptors that modulate immune response and tissue repair (Damani et al. 2011). Upon activation, microglia prime and activate autoreactive T‐cells by upregulating MHC molecules and expressing co‐stimulatory molecules like CD80 and CD86 (Guo et al. 2022; Jurga, Paleczna, and Kuter 2020). Additionally, microglia release cytokines that attract T‐cells to the retinal parenchyma, thereby contributing to immune responses in RD diseases (Ramirez et al. 2017). Microglia, as the primary immune cells of the retina, function as true APCs. They can express both MHC Class I and II molecules, allowing them to present antigens to both CD8+ and CD4+ T cells, respectively (Hayes, Woodroofe, and Cuzner 1987; Wolf et al. 2018).

While microglia are the primary immune cells of the retina, Müller cells serve as the macroglia, acting as the structural and functional equivalents by responding to retinal stress and injury, modulating immune responses, and maintaining retinal homeostasis (Reichenbach and Bringmann 2020). Müller cells span the entire depth of the retina, contributing to light transmission and maintaining homeostasis under physiological conditions (Szabó, Erdei, and Maák 2022; Yoshimoto et al. 2023). In response to RD, they exhibit reactivity to protect the retina from further damage and promote repair following pathological insult. Reactive gliosis includes morphological, biochemical, and physiological changes, which vary depending on the type and degree of the injury (Graca, Hippert, and Pearson 2018). Reactive Müller cells are characterized by altered expression of both specific and nonspecific markers. Glial fibrillary acidic protein (GFAP) is widely recognized as a marker of Müller cell gliosis, typically upregulated in response to retinal injury; however, it is not entirely specific, as it can also be expressed in reactive astrocytes (Bringmann et al. 2006). In contrast, glutamine synthetase (GS) is generally considered a more specific marker for Müller cells, crucial for glutamate metabolism. Its expression may vary during gliosis—either maintained or reduced—depending on the severity of the retinal insult (Reichenbach and Bringmann 2013). Together, the expression patterns of GFAP and GS reflect the complex responses of Müller cells to retinal diseases and injuries. Simultaneously, Müller cells effectively express both MHC Class I and II molecules, enabling them to actively interact with CD4^+^ and CD8^+^ T‐cells (Lorenz et al. 2021). While not classical APCs, Müller cells can express both MHC Class I and II molecules under certain conditions, particularly during retinal stress or injury. This allows them to potentially interact with both CD8+ and CD4+ T cells, though their capacity for providing full T cell activation signals may differ from professional APCs like microglia (Bringmann et al. 2006; Chen et al. 2022).

In addition to Müller cells, astrocytes play a significant role in retinal immune and vascular regulation. Located in close association with the superficial vascular plexus, astrocytes help maintain blood‐retinal barrier integrity, modulate vascular tone, and respond to inflammatory cues within the retinal environment. In neuroinflammatory conditions such as glaucoma, astrocytes undergo reactive changes that mirror aspects of Müller cell gliosis, including upregulation of extracellular matrix proteins, increased release of pro‐inflammatory cytokines, and enhanced antigen presentation capabilities (Salkar et al. 2024). These responses underscore the active role of astrocytes in immune surveillance and inflammation, particularly in regions surrounding retinal vessels, where they serve as critical mediators of neurovascular interactions and immune responses in the retina (Salkar et al. 2024; Shinozaki, Kashiwagi, and Koizumi 2023). Like Müller cells, astrocytes can express both MHC Class I and II molecules under pathological conditions (Rostami et al. 2020; Song et al. 2016), potentially interacting with T cells, though their role as APCs may be limited.

Thus, microglia and Müller cells can potentially interact with different subsets of T‐cells through the expression of MHC molecules during RD diseases. However, how glial cells coordinate the T‐cell response to retina damage remains unknown. There is a compelling need to understand the specific role of glial cells in T‐cell modulation during RD and how their interaction governs complex cell–cell interactions that, in turn, may influence the retina's ability to recover from injury. While all nucleated retinal cells can participate in immune surveillance through MHC Class I presentation, the specific antigen‐presenting capabilities of microglia as true APCs, and the potential APC‐like functions of Müller cells and astrocytes under pathological conditions, warrant further investigation.

Here, we utilized a well‐established laser‐induced mouse model of focal retinal degeneration, which creates precise and reproducible damage to photoreceptors in the outer retinal layers (Conedera, Pousa, et al. 2021; Khan et al. 2023; Stalker 2020). This model, combined with retrospective studies on human retinas and in vitro experiments, allowed us to determine the role of glial cells in the T‐cell response to retinal damage. Such information is fundamental for understanding retinal disorders and developing targeted therapeutic strategies that safely modulate the immune response to RD and improve outcomes.

## 2 Results

### 2.1 Resident Immune Cells Act as APCs in Response to Injury in the Retina via the MHC Class II Pathway

Microglia and resident macrophages represent the main APC inside the retinal parenchyma (Schetters et al. 2017). The contribution of APCs in a disease like uveitis is well established, while the role of T‐cells in degenerative diseases, such as AMD, is poorly understood (Sutter and Crocker 2022). We utilized a focal laser injury model that mimics key features of retinal degeneration, including photoreceptor degeneration, local inflammatory responses, leukocyte recruitment, as well as glial reactivity and scarring, mirroring observations in damaged retinas in humans (Conedera, Quintela Pousa, et al. 2021).

**FIGURE 1:**
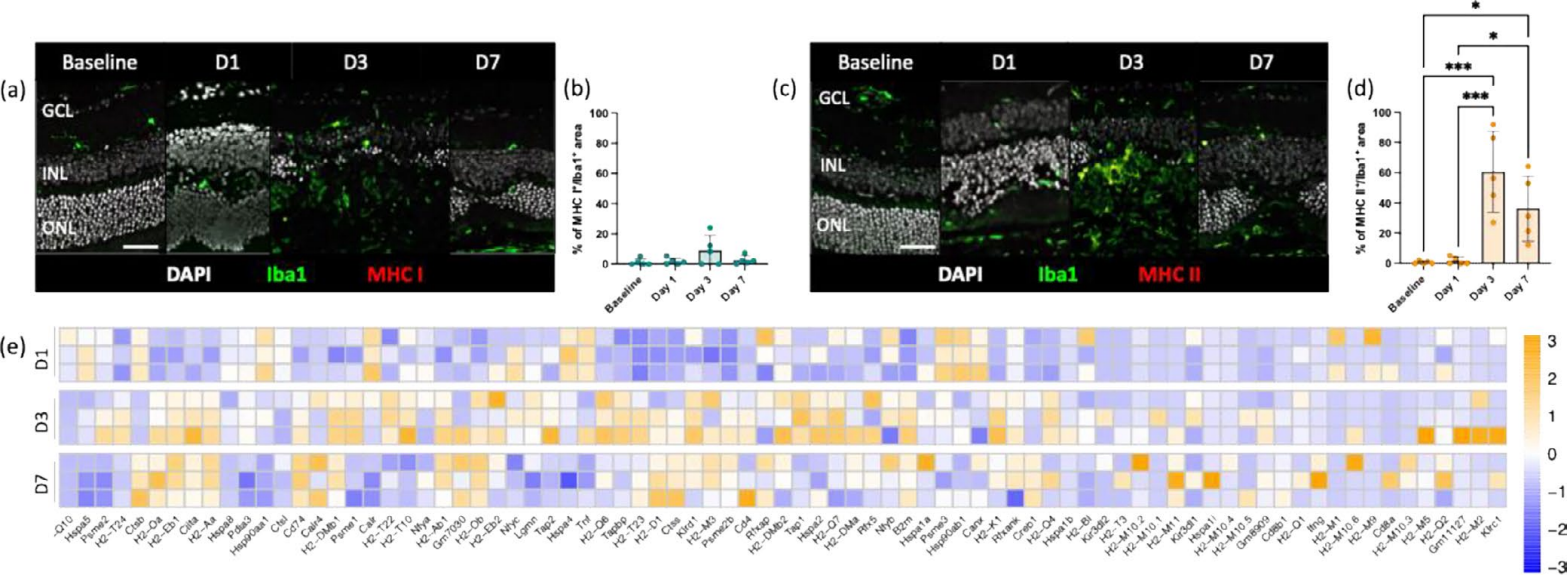
Retinal injury triggers microglial MHC II expression and CD4^+^ T‐cell signaling upregulation. (a–d) Analysis of MHC expression by microglia after injury using a microglia marker (Iba1) and MHC Class I (MHC I) or Class II (MHC II) marker. (a) Representative sections stained for Iba1 (green) and MHC I (red). Scale bars equals 100 μm. GCL, ganglion cells layer; INL, inner nuclear layer; ONL, outer nuclear layer. (b) Quantification of the MHC I^+^Iba1^+^ area on total Iba1^+^ area per lesion before injury (baseline) and at pre‐defined time points (Days 1, 3, and 7). Significant differences between baseline and the different time points were determined by using a post hoc Bonferroni one‐way ANOVA test (*n* = 5). (c) Representative sections stained for Iba1 (green) and MHC II (red). Scale bars equals 100 μm. GCL, ganglion cells layer; INL, inner nuclear layer; ONL, outer nuclear layer. (d) Quantification of the percentages of the MHC II^+^Iba1^+^ area on total Iba1^+^ area per lesion before injury (baseline) and at pre‐defined time points (Days 1, 3, and 7). Significant differences (*p < 0.1 and ***p < 0.001) between baseline and the different time points were determined by using a post hoc Bonferroni one‐way ANOVA test (*n* = 5). (e) Heatmaps of differentially expressed APC‐related genes in Csfr1^EGFP^ cells, represented as *z*‐scores. Genes were selected from KEGG pathways (mmu04612).

We assessed the expression of MHC molecules in resident phagocytic cells (Iba1^+^) as a key characteristic of APCs (Figure 1a–d). Before injury and 24 h post‐injury, Iba1^+^ cells showed negative staining for both classes of MHC molecules (Figure 1b,d). However, starting from Day 3 post‐injury, we observed an increase in their MHC levels (Figure 1a–d). Although a 9% increase in MHC I expression was detected in Iba1^+^ cells, it did not significantly differ from baseline (*p* value = 0.15, Figure 1a,b). By Day 7, only 2% of resident phagocytic cells expressed MHC I, similar to the level found before injury (Figure 1b). In contrast, MHC II protein levels significantly increased compared with baseline and Day 1 on Days 3 and 7 post‐injury (Figure 1c,d). We found that 60% of Iba1^+^ cells were MHC II positive on Day 3, with only 36% remaining positive on Day 7; however, there was no statistical difference between these two time points (Figure 1c,d).

To validate our immunofluorescence data, we conducted a transcriptome analysis of phagocytic cells expressing the colony‐stimulating factor 1 receptor (Csf1r) before and after injury at specified time points (Days 1, 3, and 7). Using RNAseq from Csf1r^+^ cells, we identified more than 90 genes related to antigen processing and presentation with significant fold‐changes (*p* value < 0.05; Figure 1e and Figure S2). Interestingly, the expression of these genes was predominantly upregulated on Days 3 and 7 post‐injury concomitant with the MHC II expression in Iba1^+^ cells (Figure 1e). Among the significantly upregulated transcripts, we observed histocompatibility 2 Class II antigen E beta2 (H2‐Eb2), HLA Class II histocompatibility antigen gamma chain (Cd74) and Class II MHC transactivator (Ciita; Figure 1e and Figure S2). These genes have previously been shown to play crucial roles in the MHC II pathway, contributing to the antigen presentation process essential for adaptive immune responses (Accolla et al. 2019; Cloutier, Fortin, and Thibodeau 2021; Logunova et al. 2023). In particular, H2‐Eb2 facilitates the presentation of peptides to CD4^+^ T‐cells, while Cd74 aids in stabilizing and directing newly synthesized MHC II molecules. Ciita regulates MHC II gene transcription by binding to their promoters and recruiting transcriptional machinery, thereby essential for the expression of MHC II molecules on the surface of APCs.

These data indicate that resident phagocytic cells upregulated MHC II and its pathway, suggesting their ability to present antigens to CD4^+^ helper T‐cells.

**FIGURE 2:**
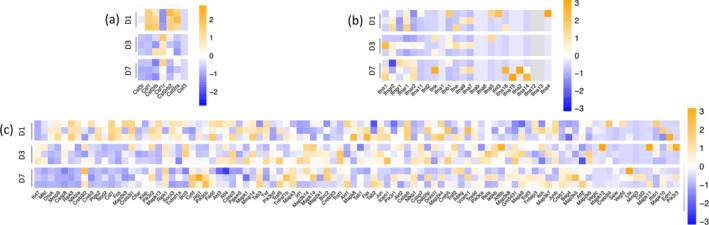
Differential gene expression profiles of CSF receptors, interferon receptor–ligand genes, and TNF pathway genes in microglia after laser injury. (a) Heatmaps of differentially expressed genes that encode receptors for CSFs in Csfr1^EGFP^ (microglia), represented as z‐scores. Data are expressed as fold‐changes between different time points (Days 1, 3, and 7) compared with negative controls (Csfr1^EGFP^ cells from uninjured retinas). (b) Heatmaps of differentially interferon receptor–ligand genes in Csfr1^EGFP^ (microglia), represented as *z*‐scores. Data are expressed as fold‐changes between different time points (Days 1, 3, and 7) compared with negative controls (Csfr1^EGFP^ cells from uninjured retinas). (c) Heatmaps of differentially expressed genes of the TNF signaling pathway in Csfr1^EGFP^ (microglia), represented as *z*‐scores. Genes were selected from KEGG pathways (mmu04668).

Furthermore, our RNA sequencing data revealed an upregulation of certain cytokines and pro‐inflammatory molecules known to enhance the expression of MHC molecules during the injury response 24 h after injury (Figure 2a–c and Figure S3). Csf1r, however, was significantly upregulated on Days 3 and 7 compared with baseline (Figure 2a and S3). These findings provide evidence that CSF signaling may promote immune cell differentiation and activation, potentially influencing their capacity to effectively present antigens via the MHC II pathway. Cytokines such as interferons (IFNs) and tumor necrosis factor alpha (TNF‐α) are known to enhance the expression of MHC molecules, thereby facilitating antigen presentation and immune responses during inflammation or immune activation. However, we did not find a statistically significant difference in the expression of either IFN Type I or TNF‐α pathway genes compared with basal levels. Additionally, we observed a major upregulation in chemokine expression primarily on Day 1 post‐injury, which may drive initial T‐cell migration, suggesting that while T‐cell migration could be initially driven by chemokines, later changes involving MHC molecules might be more closely associated with cytokines (Figure 2 and Figure S3).

These data indicates an enhanced activation and differentiation of resident phagocytic cells, potentially influencing their ability to effectively present antigens. Moreover, our findings suggest that IFN Type I and TNF‐α may not play primary roles as mediators in this context, though this conclusion is limited by the low sample size and considerable interindividual variability observed.

### 2.2 Müller Cells Upregulate the MHC Class I Pathway in Response to Injury in the Retina

Apart from microglia, Müller cells are capable of inducing both MHC Class I and MHC Class II with co‐stimulatory molecules in vitro (Schmalen et al. 2021). However, there is limited literature on their ability in response to retinal damage in murine models.

**FIGURE 3:**
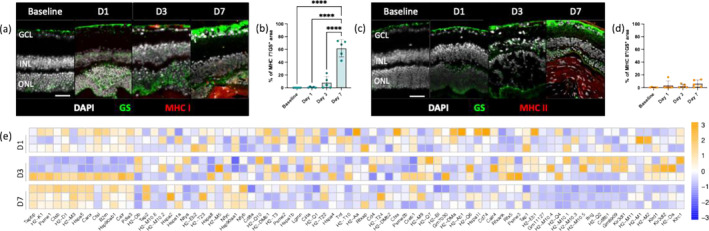
Retinal injury induces the expression of MHC Class I in Müller glia and upregulates CD8 T‐cell signaling. (a–d) Analysis of MHC proteins expression by Müller cells after injury using a Müller cell marker (GS) and MHC Class I (MHC I) or Class II marker (MHC II). (a) Representative sections stained for GS (green) and MHC I (red). Scale bars equals 100 μm. GCL, ganglion cells layer; INL, inner nuclear layer; ONL, outer nuclear layer. (b) Quantification of the percentages of the MHC I + GS+ area on total GS+ area per lesion before injury (baseline) and at pre‐defined time points (Days 1, 3, and 7). Significant differences (****p < 0.0001) between baseline and the different time points were determined by using a post hoc Bonferroni one‐way ANOVA test (*n* = 5). (c) Representative sections stained for GS (green) and MHC II (red). Scale bars equals 100 μm. GCL, ganglion cells layer; INL, inner nuclear layer; ONL, outer nuclear layer. (d) Quantification of the percentages of the MHC II + GS+ area on total GS+ area per lesion before injury (baseline) and at pre‐defined time points (Days 1, 3, and 7). Significant differences between baseline and the different time points were determined by using a post hoc Bonferroni one‐way ANOVA test (*n* = 5). (e) Heatmaps of differentially expressed APC‐related genes in Rlbp1GFP cells, represented as *z*‐scores. Genes were selected from KEGG pathways (mmu04612).

Therefore, we assessed the expression of MHC molecules in Müller glia (GS^+^) in response to focal injury to the retinal parenchyma (Figure 3a–d). Similar to our observations in microglia, GS^+^ cells were negative for both classes of MHC molecules before injury and at 24 h post‐injury (Figure 3b,d). We observed that on Day 3 post‐injury, MHC I protein levels in Müller cells showed a slight increase, with 8% positivity. However, this increase only reached statistical significance on Day 7 post‐injury, when over half of GS^+^ cells expressed MHC I (62%). In contrast, MHC II protein levels in Müller cells remained similar to basal levels throughout the injury response, showing a nonsignificant increase on Day 7 post‐injury compared with baseline (*p* value = 0.27, Figure 3c,d).

To investigate whether Müller cells actively participate in the injury response as an atypical APCs, we conducted a transcriptome analysis of retinaldehyde‐binding protein 1 (Rlbp1)‐positive cells. In the context of retinal injury or disease, Müller cell proliferation often occurs in response to damage as part of the retinal repair process, potentially leading to the formation of a glial scar (Conedera, Pousa, et al. 2021; Karl et al. 2008; Liu et al. 2013). Thus, we analyzed only Rlbp1^+^ cells that re‐enter the cell cycle. RNA sequencing was performed in reactive Müller cells before and after injury at specified time points (Days 1, 3, and 7). We observed that most of the genes related to antigen processing and presentation were predominantly upregulated 7 days after injury, coinciding with the expression of MHC I in GS^+^ cells (Figure 3e). Among the significantly upregulated transcripts, we observed proteasome activator subunit 1 (Psme1), transporter 2 ATP binding cassette subfamily b member (Tap2), TAP binding protein (Tapbp) and calnexin (Canx; Figure 3e and Figure S5). Psme1 assists in the generation of peptides for MHC Class I presentation, Tap genes facilitate the transport of peptides for MHC Class I loading, and Canx aids in the folding and assembly of MHC Class I molecules. These genes collectively play important roles in the MHC Class I antigen processing and presentation pathway, contributing to the immune response by presenting intracellular antigens to cytotoxic T‐cells.

We hypothesize that Müller glia may function as atypical APCs by upregulating MHC I and its associated pathway, potentially presenting antigens to CD8^+^ cytotoxic T cells.

**FIGURE 4:**
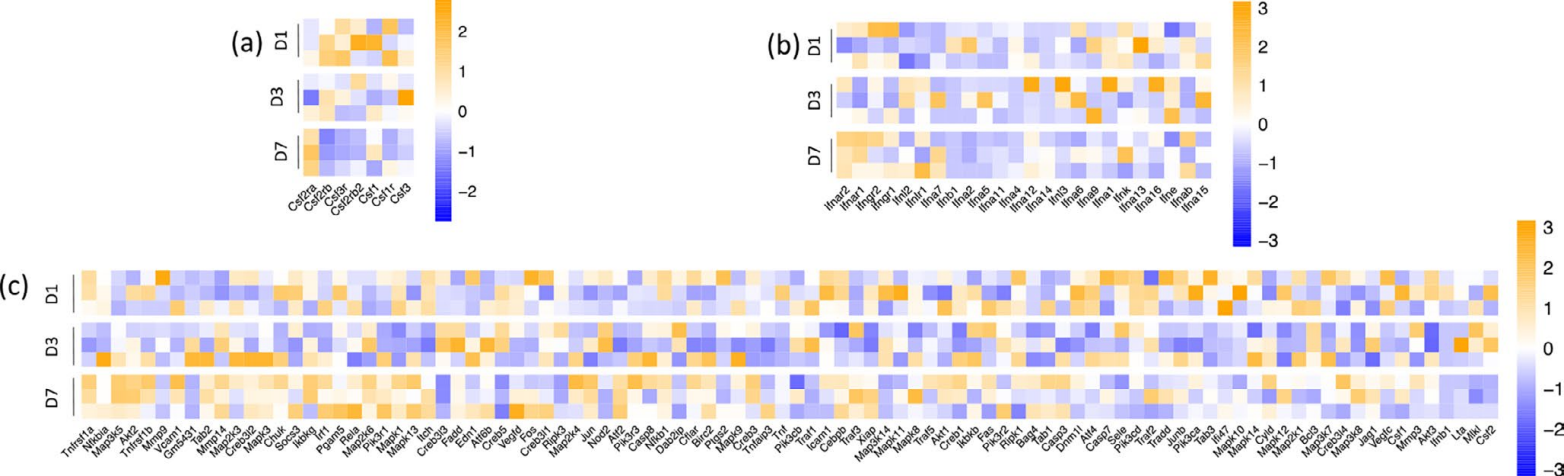
Differential gene expression profiles of CSF receptors, interferon receptor–ligand genes, and TNF pathway genes in Müller glia after laser injury. (a) Heatmaps of differentially expressed genes that encode receptors for CSFs in Rlbp1^GFP^ cells (Müller glia), represented as *z*‐scores. Data are expressed as fold‐changes between different time points (Days 1, 3, and 7) compared with negative controls (Rlbp1^GFP^ cells from uninjured retinas). (b) Heatmaps of differentially interferon receptor‐ligand genes in Rlbp1^GFP^ cells (Müller glia), represented as *z*‐scores. Data are expressed as fold‐changes between different time points (Days 1, 3, and 7) compared with negative controls (Rlbp1^GFP^ cells from uninjured retinas). (c) Heatmaps of differentially expressed genes of the TNF signaling pathway in Rlbp1^GFP^ cells (Müller glia), represented as *z*‐scores. Genes were selected from KEGG pathways (mmu04668).

Additionally, our RNA sequencing data showed an elevation in certain signaling molecules associated with inflammation, known for their capacity to act on MHC pathways during injury response (Figure 4a–c and S6). Müller cells have an increased gene expression of CSF signaling similar to what observed in the resident immune cell response. However, reactive macroglia upregulated only Csf2ra 7 days post injury, rather than Csf1r (Figure 4a and Figure S6). The expression of Csf2ra in Müller cells may be connected to their immunomodulatory functions and their potential role in antigen presentation via MHC I molecules, contributing to immune surveillance and responses within the retina. Interestingly, we found significant differences in IFN Type I receptor expression, especially Ifnar1 and 2, and the ligand Ifna7 compared with their basal level (Figure 4a and Figure S4). Activation of Ifnar1 and Ifnar2 initiates a signaling cascade that leads to the upregulation of genes responsible for the synthesis and assembly of MHC I molecules, as well as components involved in the processing and presentation of antigens. In addition, TNF‐α pathway is upregulated in response to injury (Figure 4a and Figure S5), enhancing the expression of MHC molecules and facilitating antigen presentation during inflammation. Unlike microglia, Müller cells did not show a major upregulation in chemokine expression, suggesting that T‐cell migration driven by Müller cells may instead rely on MHC upregulation.

Our findings suggest Müller cells actively participate in retinal adaptive immunity by potentially presenting antigens to CD8^+^ T‐cells. Müller glia may upregulate the MHC Class I pathway through IFNs and TNF‐α pathway.

### 2.3 Glial Cell‐Mediated Antigen Presentation in Human Retinas During Degeneration

Several studies have also shown that glial cells are capable of acting APCs in preclinical models of retinal diseases; however, little is known about their role as APCs in humans.

**FIGURE 5:**
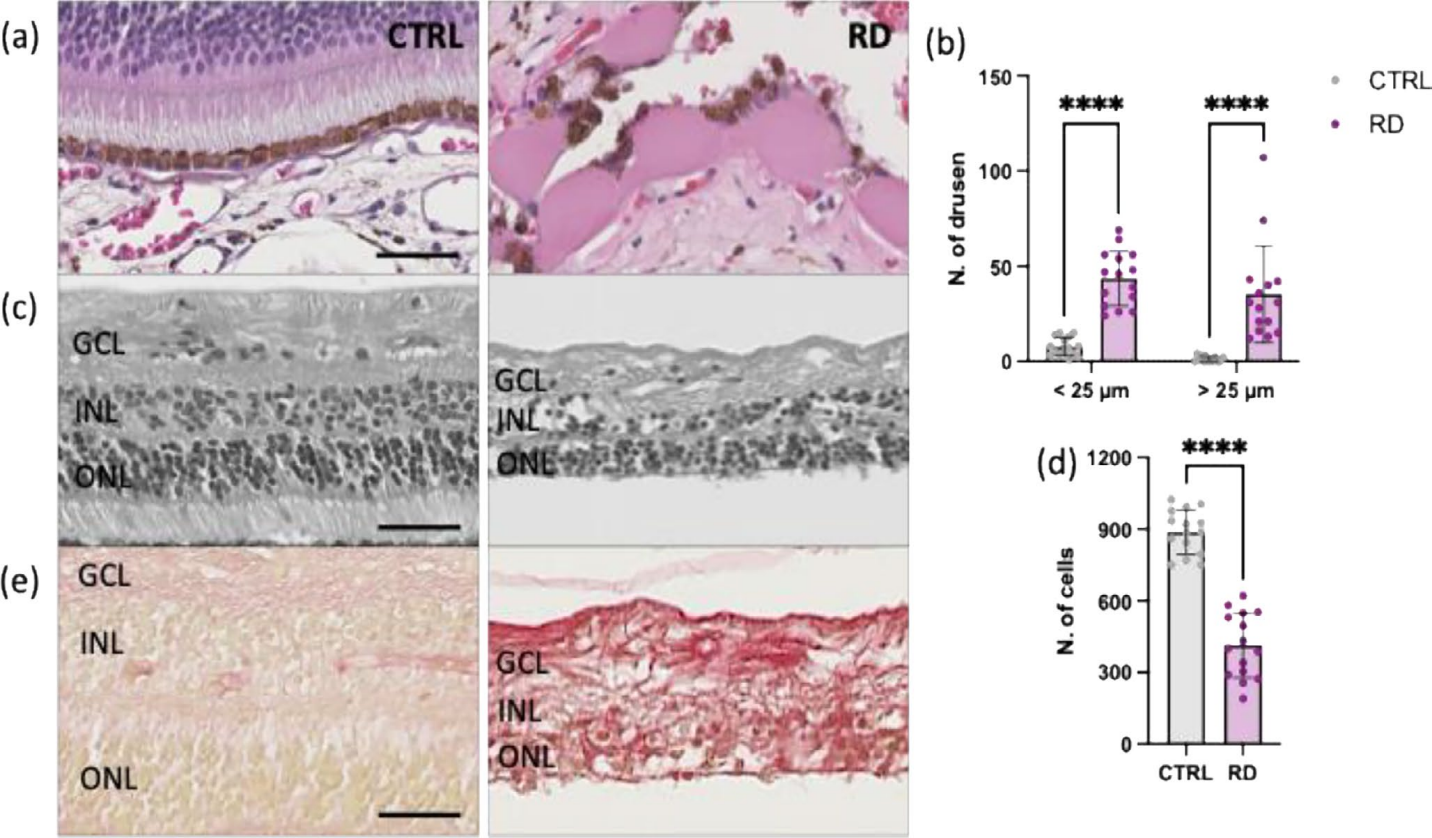
Pathological examination of ocular tissue from human donors' eyes. (a–d) H&E staining of human retinas showing presence of drusen and its integrity. (a) Representative detailed view of healthy cuboidal RPE (CTRL) and retinas presenting drusen (RD). Scale bars equals 50 μm. (b) Quantification of drusen size, categorized as either ≤ 25 μm or > 25 μm in width. Significant differences (****p < 0.0001) between CTRL and RD groups were determined by using two‐tailed Mann–Whitney test analysis (*n* = 15). (c) Representative detailed view of healthy retinal structure (CTRL) and atrophic retinas (RD). Scale bars equals 50 μm. GCL, ganglion cells layer; INL, inner nuclear layer; ONL, outer nuclear layer. (d) Quantification of cell nuclei per retina. Significant differences (****p < 0.0001) between CTRL and RD groups were determined by using two‐tailed Mann–Whitney test analysis (*n* = 15). (e) Representative sections stained with Picro Sirius red staining for histological visualization of collagen fibers and retinal fibrosis. Scale bars equals 50 μm. GCL, ganglion cells layer; INL, inner nuclear layer; ONL, outer nuclear layer.

Therefore, we first analyzed histopathological features of retinal degeneration in ocular tissue from human donor eyes by investigating the presence of drusen and structural changes in the retinal tissue (Figure 5). Using H&E staining, we identified 15 specimens exhibiting healthy cuboidal retinal pigment epithelium (RPE). These cells displayed a uniform shape, with centrally located nuclei and abundant melanin pigment granules in the cytoplasm, giving them a brownish appearance. The borders between adjacent RPE cells were well‐defined, and the basal surface of RPE cells lay on Bruch's membrane (Figure 5a). These retinal samples exhibited a normal multilayered structure with distinct cellular arrangements and were categorized as the (Figure 5a,c,d). Conversely, 15 specimens showed drusen, which typically appeared as discrete, round, or oval‐shaped deposits located between the RPE and Bruch's membrane. Drusen often presented as eosinophilic (pink‐staining) structures, with sizes ranging from 25 to 125 μm. Specimens with drusen exhibited a thinner retina, reduced cellular density, and structural disorganization compared with the CTRL group. This ongoing degeneration was evident from the loss of cellular components observed in the H&E‐stained sections, leading us to classify these specimens as the RD group (Figure 5a,c,d). To further confirm the pathological state of the RD retinas compared with CTRL, we performed Picro Sirius red staining to visualize tissue fibrosis. As expected, the RD group displayed fibrotic retinas with prominent red collagen fibers, often arranged in irregular patterns or bundles. In contrast, CTRL specimens exhibited a pale pink coloration, indicating the absence of fibrosis or pathological changes characteristic of a healthy physiological state (Figure 5e).

**FIGURE 6:**
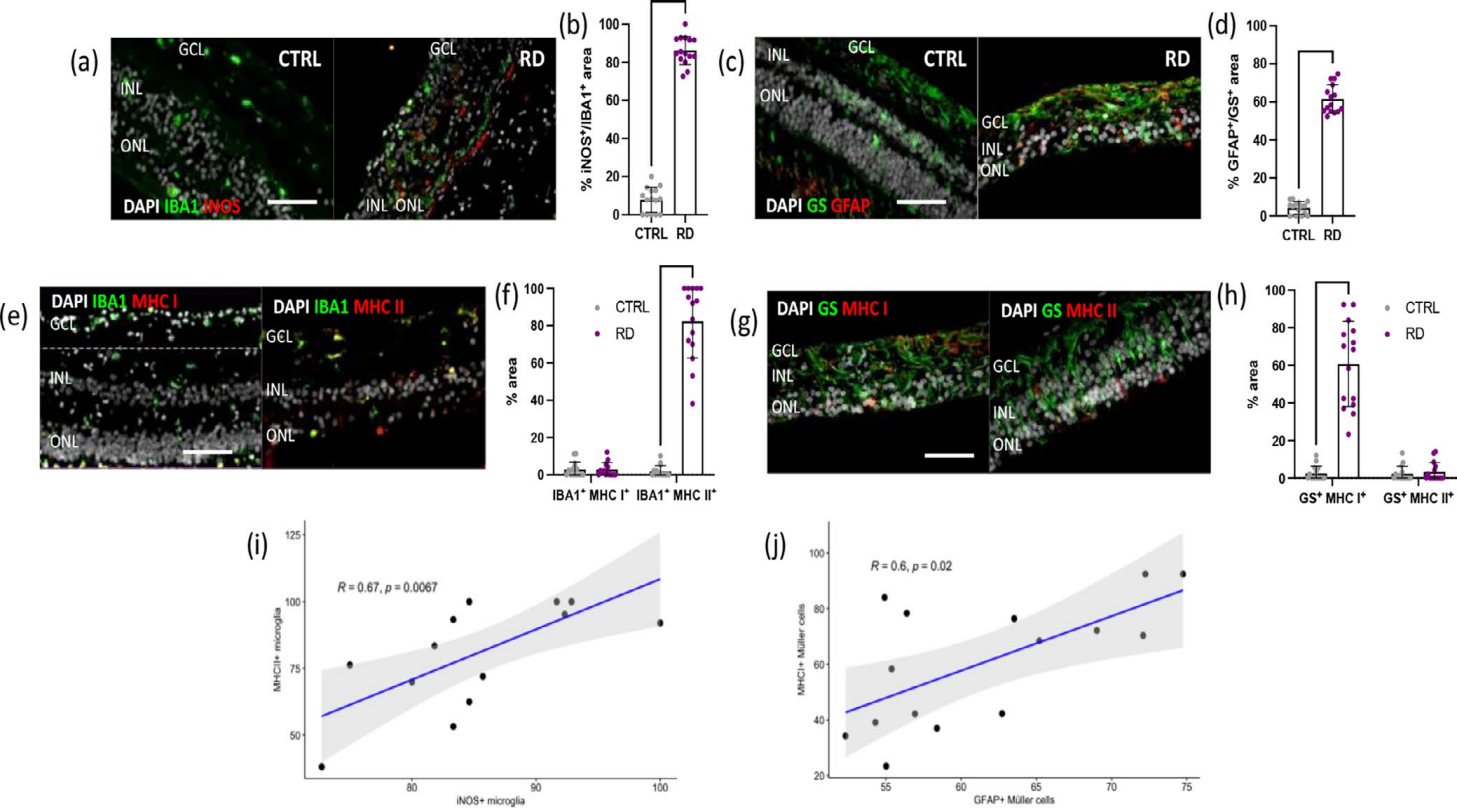
Glial responses correlate MHCs in human retina during degeneration. (a, b) Analysis of pro‐inflammatory phenotype in microglia in healthy (CTRL) and degenerated retinas (RD) using a microglia marker (IBA1) and a pro‐inflammatory marker (iNOS). (a) Representative sections stained for Iba1 (green) and iNOS (red). Scale bars equals 100 μm. GCL, ganglion cells layer; INL, inner nuclear layer; ONL, outer nuclear layer. (b) Quantification of the percentages of iNOS^+^IBA1^+^ area on total IBA1^+^ area in CTRL and RD retinas. Significant differences (****p < 0.0001) between CTRL and RD were determined by using two‐tailed Mann–Whitney test analysis (*n* = 15). (c, d) Analysis of Müller cell reactivity in healthy (CTRL) and degenerated retinas (RD) using a Müller cell marker (GS) and a reactivity marker (GFAP). (c) Representative sections stained for GS (green) and GFAP (red). Scale bars equals 100 μm. GCL, ganglion cells layer; INL, inner nuclear layer; ONL, outer nuclear layer. (d) Quantification of the percentages of the GFAP^+^GS^+^ area on total GS^+^ area in CTRL and RD retinas. Significant differences (****p < 0.0001) between CTRL and RD were determined by using two‐tailed Mann–Whitney test analysis (*n* = 15). (e–h) Analysis of MHC proteins by glia in degenerated retinas. (e) Representative sections stained for Iba1 (green) and MHC I or II (red). Scale bars equals 100 μm. GCL, ganglion cells layer; INL, inner nuclear layer; ONL, outer nuclear layer. (f) Quantification of the percentages of the MHC I^+^ IBA1^+^ and MHC II^+^ IBA1^+^ area on total IBA1^+^ area in CTRL and RD retinas. Significant differences (****p < 0.0001) between CTRL and RD were determined by using two‐tailed Mann–Whitney test analysis (*n* = 15). (g) Representative sections stained for GS (green) and MHC I or II (red). Scale bars equals 100 μm. GCL, ganglion cells layer; INL, inner nuclear layer; ONL, outer nuclear layer. (h) Quantification of the percentages of the MHC I^+^GS^+^ and MHC II^+^GS^+^ area on total GS^+^ area in degenerated retinas. Significant differences (****p < 0.0001) between CTRL and RD were determined by using two‐tailed Mann–Whitney test analysis (*n* = 15). (i) Spearman correlation between MHC II^+^ IBA1^+^ cells with iNOS^+^ IBA1^+^ cells in the degenerated retinas. (j) Spearman correlation between MHC I^+^GS^+^ cells with GFAP^+^GS^+^ cells in the degenerated retinas.

Then, we assessed whether resident phagocytic cells (Iba1^+^) and Müller glia (GS^+^) exhibited characteristics of APCs upon activation in the diseased retina (Figure 6a,b). Iba1^+^ cells were exclusively iNOS^+^ in RD specimens (Figure 6a,b), indicating their activation during degenerative processes and their polarization into a pro‐inflammatory state. Additionally, we observed reactive Müller glia in RD samples. During RD, over 60% of GS^+^ cells co‐expressed GFAP, compared with only 4% of GFAP^+^GS^+^ cells found in the CTRL group. The presence of GFAP^+^ Müller glia during RD indicates their reactive and gliotic state compared with the quiescent state in healthy conditions.

In order to define the impact of RD on glia APCs, we assessed the expression of MHC molecules in phagocytic cells and Müller glia in the retinal parenchyma comparing CTRL and RD group. Concomitant with the upregulation of iNOS in microglia and GFAP in Müller glia, we found the expression of MHC proteins only in degenerated retinas. Interestingly, different glial type expressed different classes of MHC. MHC I expression was detected in less than 3% of Iba1^+^ cells in both CTRL and RD groups. While only 1.8% of cells in CTRL specimen expressed MHC II, we observed a significant increase to 82% of phagocytic cells positive for MHC II in RD retinas. In Müller glia, an opposite trend was observed, as GS^+^ cells were negative for MHC II in CTRL and RD retinas. However, 60% of Müller glia were positive for MHC I exclusively in diseased retinas.

This analysis revealed that phagocytic cells expressed iNOS and MHC I in the degenerated retina, while Müller cells showed positivity for GFAP and MHC II. Consequently, we investigated the relationship between glial reactivity and their expression of MHC molecules through correlation analysis (Figure S7). Statistical analysis of our data confirmed a significant positive correlation between iNOS and MHC I in Iba1^+^ cells, as well as between GFAP and MHC II in GS^+^ cells, highlighting the distinct involvement of microglia and Müller cells in the immune response within the retinal microenvironment under pathological conditions. Furthermore, our results suggest that the observed upregulation of MHC I and MHC II on microglia and Müller cells may facilitate antigen presentation to CD8^+^ and CD4^+^ T‐cells, respectively, thereby modulating the adaptive immune response differently.

**FIGURE 7:**
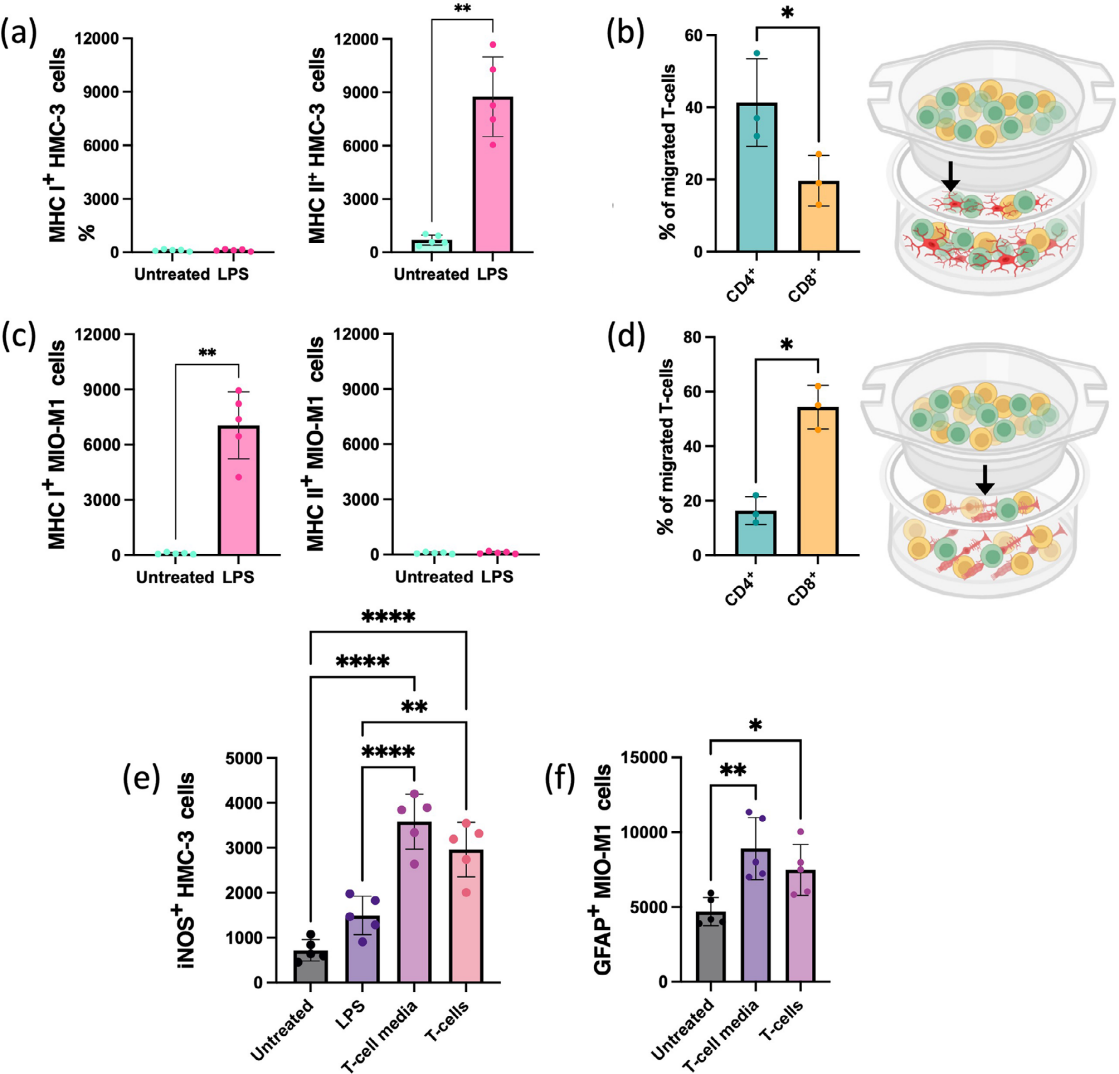
Reciprocal interplays between glia and T‐cells in vitro. (a) Quantification of the MHC I^+^ and MHC II^+^ HMC‐3 cells untreated and LPS treated. Significant differences (**p < 0.1) between untreated and LPS‐treated cells were determined using two‐tailed Mann–Whitney test analysis (*n* = 5). (b) Mean ± SD of the percentages of CD4^+^CD3^+^ and CD8^+^CD3^+^ cells migrated toward microglia. Significant differences (*p < 0.1) between CD4^+^CD3^+^ and CD8^+^CD3^+^ cells migrated were determined using two‐tailed Mann–Whitney test analysis (*n* = 3). (c) Quantification of the MHC I^+^ and MHC II^+^ MIO‐M1 cells untreated and LPS treated. Significant differences (**p < 0.1) between untreated and LPS‐treated cells were determined using two‐tailed Mann–Whitney test analysis (*n* = 3). (d) Mean ± SD of the percentages of CD4^+^CD3^+^ and CD8^+^CD3^+^ cells migrated toward Müller cells. Significant differences (*p < 0.1) between CD4^+^CD3^+^ and CD8^+^CD3^+^ cells migrated were determined using two‐tailed Mann–Whitney test analysis (*n* = 3). (e) Quantification of the iNOS^+^ HMC‐3 cells untreated, treated with LPS, T‐cell media or co‐cultured with T‐cells. Significant differences (**p < 0.01 and ****p < 0.0001) between conditions were determined by using a post hoc Bonferroni one‐way ANOVA test (*n* = 5). (f) Quantification of the GFAP^+^ MIO‐M1 cells untreated, treated with T‐cell media or co‐cultured with T‐cells. Significant differences (*p < 0.1 and **p < 0.001) between conditions were determined by using a post hoc Bonferroni one‐way ANOVA test (*n* = 5).

In vitro experiments were conducted to corroborate reciprocal impacts between glia and T‐cells. For these experiments, we used the human‐derived microglial cell line HMC‐3 and the human‐derived, spontaneously immortalized Müller cell line MIO‐M1. Primary human T‐cells were isolated from buffy coats obtained through the Interregional Blood Bank Bern. T‐cells were then co‐cultured with either HMC‐3 or MIO‐M1 cells to investigate bidirectional cellular interactions and signaling responses. To validate glial activation, we first analyzed MHC I and II expression in HMC‐3 microglia, both untreated and after LPS treatment. LPS‐treated HMC‐3 cells significantly upregulated MHC II (but not MHC I), showing a pattern comparable to ex vivo analyses (Figures 6 and 7a). Migration assays revealed that CD4^+^ T‐cells migrated more frequently toward HMC‐3 microglia than CD8^+^ T‐cells, with approximately double the number of CD4^+^ cells transmigrating (Figure 7b). Similarly, we assessed MHC I and II expression in MIO‐M1 Müller cells under untreated and LPS‐treated conditions. LPS‐treated MIO‐M1 cells showed an increase in MHC I expression but no significant change in MHC II, aligning with ex vivo observations (Figures 6 and 7c). Migration assays for Müller cells showed that CD8^+^ T‐cells migrated toward MIO‐M1 at a rate approximately three times higher than CD4^+^ T‐cells (Figure 7d).

Finally, we incubated HMC‐3 and MIO‐M1 cells with activated T‐cells or their media to analyze their effects on glial cells. As expected, a higher number of LPS‐treated microglia expressed iNOS compared with untreated cells. Interestingly, this expression increased further when incubated with activated T‐cells or their media, suggesting that activated T‐cells induced a pro‐inflammatory phenotype in HMC‐3 microglia (Figure 7c). Additionally, cultured Müller cells expressed GFAP exhibiting reactivity, but when exposed to activated T‐cells or their media, this expression increased even more, confirming the impact of activated T‐cells on Müller glia reactivity (Figure 7d).

Overall, our ex vivo and in vitro data demonstrate strong and specific T‐cell‐glial cell interactions. Different T‐cell subpopulations are recruited by various types of glia, which in turn become more active upon interaction with T‐cells.

## 3 Discussion

Since the 1980s, retinal diseases have been intricately linked to immune system involvement (Dumonde et al. 1985; Keltner, Roth, and Chang 1983). Numerous clinical trials have focused on modulating retinal inflammation to promote tissue regeneration, but achieving consistent treatment success has proven challenging. The underlying causes of these failures are unclear (Kim, Kim, and Oh 2021).

In recent years, diverse paradigms concerning retinal immunology have been challenged, including the discovery of glial cells acting as APCs and the role of T‐cells in driving neurodegeneration (Grüntzig and Hollmann 2019; Sutter and Crocker 2022; Zhang and Jiang 2023). Once retinal tissue is damaged, eye‐derived antigens drain into the lymph nodes to activate T‐cells, which are recruited in the retinal parenchyma. However, we still have limited knowledge about the functional role of glial cells as APCs within the damaged retinal tissue (Schetters et al. 2017).

We used a laser coagulator to induce photoreceptor disorganization, glial proliferation and scarring, common macroscopic features of human RD diseases (e.g., AMD; Conedera, Pousa, et al. 2021). Previous studies have shown that laser‐induced injury triggers the migration of resident immune cells and macroglial reactivity specifically within the site of injury (Chidlow et al. 2013; Miller et al. 2019). Therefore, the laser‐induced injury model proves valuable for investigating glial biological processes directly within the damaged area, while using adjacent tissue as a control.

Activated microglia and resident macrophages are frequently observed in various neuropathologies and implicated in their progression (Goddery et al. 2021). Intriguingly, many neurological conditions characterized by their activation also exhibit evidence of infiltrating T‐cell populations (Behnke, Wolf, and Langmann 2020). As microglia are known to have the capability to present antigens to T‐cells, these observations have spurred further investigation into the potential role of microglia as APCs in several retinal pathologies (Biswas 2023). In line with the literature, our data from immunofluorescence and RNAseq confirmed the ability of resident immune cells to act as APCs upon laser‐induced injury. We observed an upregulation of genes and proteins related to the MHC II pathway, unlike MHC I expression, which was not significantly different from the baseline. Among the upregulated molecules, we found H2‐Eb2, Cd74, and Ciita, which are important in aiding in peptide presentation to CD4^+^ T‐cells, stabilizing MHC II molecules, and regulating MHC II gene transcription, respectively. These data showed that resident immune cells upregulated MHC II and its pathway, suggesting their ability to present antigens to CD4^+^ helper T‐cells. Supporting our results, similar interactions between MHC II^+^ resident immune cells and CD4^+^ T‐cells have been documented in other contexts, such as Tau‐mediated neurodegeneration and aging in the murine brain (Askin and Wegmann 2023; Kellogg et al. 2023; Zhang et al. 2022). In the retina, their interplay is well‐characterized only in animal models of autoimmune uveitis (Lipski et al. 2017; Okunuki et al. 2019) and little is known in RD diseases. However, we know that CD4^+^ T‐cells are recruited to the parenchyma in mice with experimental ischemic retinopathy and laser‐injuries and both models also involved glial activation (Conedera et al. 2023; Khanh Vu et al. 2020; Wagner et al. 2021). Interestingly, other recent publications implied the connection between resident immune cells and CD4^+^ T‐cells showing that the depletion of CD4^+^ T‐cells leads to the accumulation of pro‐inflammatory microglia (Llorián‐Salvador et al. 2024). Thus, resident immune cells can stimulate CD4^+^ T‐cell migration to the damaged retina as well as T‐cells can affect immune cell activation once they interplay with T‐cells. We indeed detected a significant upregulation of Csf1r 7 days post injury compared with baseline, which suggests an increased demand for CSF signaling. This data implies heightened responsiveness of immune cells to CSF1, the ligand for CSF1R, leading to their activation and proliferation. This activation enhances immune cells' ability to respond to injury signals in the retina, potentially prolonging their reactive state.

While microglia are recognized as primary APCs in the retina (Waisman and Johann 2018), it is noteworthy that macroglia‐like Müller cells, the principal glial cells of the retina, can also exhibit antigen‐presenting capabilities (Schmalen et al. 2021). This occurs in response to inflammatory conditions triggered by various pathological stimuli such as infection, trauma, or degenerative disease (Lorenz et al. 2021; Massa, Dorries, and ter Meulen 1986; Rostami et al. 2020). Under such circumstances, Müller cells upregulate antigen‐presenting molecules and actively participate in presenting antigens to T‐cells. This capability allows Müller cells to interact with the immune system and modulate immune responses within the retina. Our results confirm that Müller cells possess the machinery required for antigen presentation, as evidenced by the expression of MHC I protein. Contrarily, MHC II protein level in Müller cells remained comparable with basal levels throughout the injury response. Müller cells contribute to the injury response by acting as atypical APCs 7 days post injury, unlike resident immune cells that acquired the capacity to present antigens to T‐cells already on Day 3. The timing of microglial and macroglial responses to injury varies due to differences in their cellular properties, functions, and activation mechanisms. As sentinel cells, microglia are poised to respond rapidly to injury signals due to their constant surveillance and activation state. Microglia undergo rapid activation and migration to the injury site upon detecting injury‐associated signals, such as damage‐associated molecular patterns (DAMPs) or cytokines released by damaged cells. While Müller cells also respond to injury, their primary function is maintaining retinal homeostasis, providing metabolic support to neurons, and regulating extracellular ion and neurotransmitter levels. Furthermore, their delayed response may be related to the need for sustained changes in the retinal microenvironment, such as prolonged inflammation or cellular damage, which can activate Müller cells and induce their participation in forming a gliotic scar. Transcriptome analysis confirmed that Müller cells predominantly upregulated genes related to antigen processing 7 days after injury, coinciding with the expression of MHC I protein (Kelly and Trowsdale 2019). Psme1, various Tap genes, and Canx were significantly upregulated in Müller cells, demonstrating their ability to generate peptides for MHC Class I presentation, facilitate peptide transport for MHC Class I loading, and aid in the folding and assembly of MHC Class I molecules. Thus, these data demonstrate that Müller cells upregulate MHC Class I and the related pathway, suggesting their capacity to present antigens to CD8^+^ cytotoxic T‐cells. This differs from resident immune cells, which typically present antigens to CD4^+^ T‐cells during injury response in the retina. Although Müller cells are involved in virtually every retinal disease, their role in neuroinflammation is still poorly understood. Our results obtained from a preclinical model of RD are supported by previous studies in vitro (Lorenz et al. 2021; Schmalen et al. 2021). In these studies, they used proteomic analysis to demonstrate that Müller cells express both MHC molecules under different pro‐inflammatory stimuli. Similar to Müller cells, astrocytes can also express both MHC I and II during neurodegeneration. Infiltrated CD4^+^ T‐cells were observed interacting with MHC II‐expressing astrocytes in pathological conditions such as Parkinson's disease and multiple sclerosis (Cornet et al. 2000; Rostami et al. 2020). In vitro studies further revealed that astrocytes sustained antigen‐specific CD4^+^ T‐cell proliferation only in the presence of IFNγ, facilitated by the astrocytic expression of MHC II (Cornet et al. 2000). However, examination of the distribution of astrocytic MHC‐positive cells in relation to lesion architecture revealed distinct patterns: MHC II^+^ cells were confined exclusively to the lesion edge, while MHC I^+^ astrocytes were widely present at both the lesion edge and in the gliotic lesion center (Ransohoff and Estes 1991). These findings suggest an association between gliosis and MHC I expression, a relationship further supported by in vitro analysis (Bombeiro et al. 2017). Indeed, previous publications using the same injury model and laser setup indeed demonstrated reactive gliosis on Day 7, coinciding with our observation of MHC I pathway activation. This supports the notion that the expression of MHC molecules varies depending on the reactive status of Müller cells. However, further analyses are required to establish the direct association between gliosis and the capacity of Müller cells to function as APCs.

Glia and T‐cells interact with each other through the secretion of certain cytokines and pro‐inflammatory molecules, which have significant effects on the expression of MHC molecules (Johansson, Price, and Modo 2008; Raval et al. 1998). We indeed found an upregulation of Csf2ra in reactive Müller cells providing additional evidence of their potential role in antigen presentation via MHC I molecules, contributing to immune surveillance and responses within the retina (Saita et al. 2022). Additionally, we found significant differences in of genes related to Type I interferons and the TNF‐α pathway, which are closely linked to the regulation of MHC molecule expression and antigen presentation (Cantaert et al. 2010; Silginer et al. 2017). Previous publications showed that IFN and TNF‐α induces the expression of major histocompatibility antigens by human retinal glial cells (Chen et al. 2022; Heng et al. 2019; Mano, Tokuda, and Puro 1991). Additionally, both cytokines aggravated gliosis in the retina and brain (Hu et al. 2020; Wangler et al. 2022; Yong et al. 1991).

Inflammation and gliosis play harmful roles in RD in mice, as observed in humans (Bora et al. 2003); however, immune and glial responses can differ between the two species (Bjornson‐Hooper et al. 2022). These differences rely on variations in the extent, timing, and specific cellular mechanisms. Hence, a thorough understanding of the involvement of glia in the immune response during the progression of RD in humans is crucial for developing targeted therapies.

To achieve this, we conducted retrospective studies using formalin‐fixed and paraffin‐embedded specimens from donor eyes post‐cornea removal.

We investigated the presence of drusen and structural changes in the retinal tissue in order to classify in CTRL and RD group. This grouping reflects a well‐recognized approach in histopathology for diagnosing and understanding RD, as supported by the literature (Zhang et al. 2019). Drusen accumulate in the retina due to the buildup of waste products from retinal cells and impaired clearance mechanisms. As part of the degenerative process due to aging, the efficiency of clearing cellular debris and metabolic by‐products diminishes (Abdelsalam, Del Priore, and Zarbin 1999). This leads to the formation of drusen, which are composed of lipids, proteins, and cellular debris. The lipid components include cholesterol and phospholipids, while the proteins involve complement proteins and other immune system‐related molecules (Crabb 2014; Wang et al. 2010). This accumulation disrupts retinal function and can contribute to RD. Thus, these extracellular deposits are commonly used to characterize and differentiate between healthy and degenerative retinal tissues (Evers 3rd et al. 2023). The presence of drusen associates with the development and progression of retinal atrophy and fibrosis during RD (Chen et al. 2021). Retinal atrophy refers to the degeneration and loss of retinal cells, particularly in the macula, which is responsible for central vision. This degeneration leads to a gradual decline in visual acuity and can significantly impair a person's ability to see fine details and perform tasks such as reading and recognizing faces (Bakri et al. 2023). Fibrosis, on the other hand, involves the formation of scar tissue within the retina or beneath the RPE. Fibrosis further exacerbates vision loss by distorting the architecture of the retina and interfering with the normal function of retinal cells. Scar tissue is composed of extracellular matrix proteins such as collagen, which are deposited in response to tissue injury or inflammation (Higashijima et al. 2023). Fibrosis often occurs in advanced stages of RD and it is mostly associated with an abnormal blood vessel grow beneath the retina and leak fluid and blood (Yi et al. 2023).

We then checked if the activation state differs based on the pathological states on the human samples. Resident phagocytic cells were activated with a pro‐inflammatory phenotype only once the retina degenerated as demonstrated by the presence of iNOS^+^ Iba1^+^ cells exclusively in RD group.

Our data are in line with the literature showing that resident phagocytic cells, like microglia and perivascular macrophages, physiologically are in a resting state, monitoring the retinal environment (Murenu et al. 2022). When retinal cells start to degenerate due to factors such as drusen accumulation or oxidative stress, these phagocytic cells shift to an active state (Raoul et al. 2008). In their activated form, they produce inflammatory molecules, which further contribute to retinal damage. This inflammatory response exacerbates the degeneration of retinal cells, creating a cycle of worsening retinal health and inflammation (Rashid, Akhtar‐Schaefer, and Langmann 2019). iNOS is a reliable marker for detecting and studying the pro‐inflammatory activation of phagocytic cells during RD and reflects the transition of these cells from a resting to an activated state in response to retinal damage (Sheng et al. 2011). Additionally, other post‐mortem analyses of retinas from AMD patients have also shown elevated iNOS levels, indicating its role as a pro‐inflammatory factor contributing to disease process (Ma et al. 2009; Toma et al. 2021). Thus, iNOS is a valuable tool for understanding and characterizing the inflammatory processes involved in RD. Concurrent with iNOS expression in resident phagocytic cells, Müller glia became reactive in RD samples, as evidenced by a significant upregulation of the GFAP signal. In the CTRL retinas, only 4% of Müller glia (GS^+^) were GFAP^+^, indicating their quiescent state, consistent with their typical low‐level GFAP expression in healthy conditions (Bringmann et al. 2006). However, in RD retinas, 60% of Müller glia were GFAP^+^, reflecting their activation and gliotic response to retinal degeneration (Sarthy, Dudley, and Haldin 2018). This substantial increase in GFAP is aligned with findings that reactive gliosis, marked by GFAP upregulation, is a common response of Müller cells to retinal stress and injury, as they attempt to protect and repair retinal tissues (Zhao et al. 2015). Furthermore, the co‐expression of iNOS in resident phagocytic cells during retinal degeneration highlights the pro‐inflammatory environment, contributing to the exacerbation of retinal damage (Toma et al. 2021).

Once retina degenerates and glial cells react to tissue damage, microglia and Müller glia express MHC molecules. However, different glial type expressed different classes of MHC similarly to what we demonstrated in the murine retinas following laser‐induced injury. While phagocytic cells expressed iNOS and MHC II in the degenerated retina, Müller cells showed positivity for GFAP and MHC I.

These data implicates that different types of glial cells within the degenerated retina possess APC capabilities, as evidenced by the expression of MHC molecules. However, the specific classes of MHC molecules expressed by each glial type differ. Phagocytic cells exhibit expression of iNOS and MHC I, indicating their potential to present antigens to helper T‐cells. In contrast, Müller cells display positivity for GFAP and MHC II, suggesting their ability to present antigens to cytotoxic T‐cells. Therefore, these findings imply distinct APC abilities among glial cell types in the degenerated retina, potentially contributing to the modulation of immune responses in this context. Statistical interpretation of our ex vivo results suggested a positive and significant correlation between the reactive state of glia cells and their expression of MHC molecules. The correlation between iNOS expression and MHC II in microglia implies a coordinated response where inflammation and antigen presentation intersect. Inflammatory signals or tissue damage that induce iNOS expression in microglia may also trigger the upregulation of MHC II, potentially enhancing the ability of resident phagocytic cells to present antigens and participate in immune responses within the retina. This correlation underscores the complex interplay between inflammation and immune activation in the retina, with microglia playing a central role in both processes. Numerous studies have demonstrated the upregulation of MHC II expression in microglia under various inflammatory conditions, including neurodegenerative diseases, autoimmune disorders, and acute CNS injuries (Abellanas et al. 2019; Subbarayan et al. 2020; Wolf et al. 2018). Indeed, MHC II expression by pro‐inflammatory phagocytic cells is considered an essential aspect of their immune function and their role in the pathogenesis and regulation of neuroinflammation (Schetters et al. 2017). The correlation between GFAP expression and MHC I in Müller glia within the retina implies that the reactive state of Müller glia is linked to their ability to present antigens derived from damaged retinal cells to cytotoxic T‐cells via MHC I molecules. Therefore, the expression of both GFAP and MHC I in Müller glia may indicate their participation in immune surveillance and antigen presentation within the retina, potentially contributing to the regulation of immune responses to retinal injury or pathology. The expression of MHC I by Müller glia, particularly in the context of retinal pathology or injury, is an area of ongoing research. While Müller glia are primarily known for their roles in maintaining retinal homeostasis and providing structural support (Reichenbach and Bringmann 2013), evidence suggests that they can also exhibit immunomodulatory functions (Augustine et al. 2023), including antigen presentation. However, the specific circumstances and mechanisms regulating MHC I expression in Müller glia, as well as their functional implications, are still being elucidated. Further research is needed to fully understand the role of Müller glia‐derived MHC Class I in retinal immunity and pathology.

Finally, we aimed to further understand the link between glial responses, their APC capabilities, and different T‐cell subtypes through in vitro experiments. The migration of T‐cell subpopulations toward the glial population was cell type‐dependent: CD4^+^ T‐cells exhibited a significantly stronger response to LPS‐activated microglia compared with CD8^+^ T‐cells. LPS is a potent activator of microglia, leading to their secretion of cytokines and other factors that attract CD4^+^ T‐cells. This stronger response of CD4^+^ T‐cells is related to their role in helper functions and cytokine production in the context of immune activation (Ransohoff 2016). In contrast, CD8^+^ T‐cells preferentially migrated toward reactive Müller cells. This cell type‐dependent behavior is supported by studies on immune responses in the central nervous system and the specific roles of T‐cell subsets in neuroinflammation (Huseby et al. 2015; Sutter and Crocker 2022; Xie and Yang 2015). This directed migration was influenced by soluble factors, as confirmed by similar results obtained with supernatants from the respective glial populations. Experiments using supernatants from glial cells that contain these soluble factors often reproduce the migratory patterns observed in more complex environments, demonstrating the role of soluble mediators in directing T‐cell movement (McMahon et al. 2005; Ransohoff and Engelhardt 2012). These findings suggest optimized mechanisms for both activation‐stage and environment‐specific attributes, indicating that soluble mediators play a crucial role in the specific recruitment and movement of T‐cell subtypes toward various glial cells. This enhances our understanding of how immune cell behavior is tailored to both activation states and the microenvironment (Krummel, Bartumeus, and Gérard 2016), potentially leading to more targeted therapeutic strategies for neuroinflammatory conditions.

In summary, retinal glial cells play an active role in modulating the immune response following insults to the retinal parenchyma. Therefore, understanding the dynamics of glia‐mediated antigen presentation in the retina during degeneration is crucial for developing targeted therapeutic strategies for retinal diseases. By modulating the immune response orchestrated by glial cells toward a more beneficial one, it may be possible to mitigate RD and even potentially restore vision in affected individuals.

## 4 Materials and Methods

### 4.1 Animals

All animal experiments were approved by the local Animal Ethics Committee of the Canton of Bern, Switzerland (BE34/19) and conform to the Association for Research in Vision and Ophthalmology Statement for the Use of Animals in Ophthalmic and Vision Research. Male and female 8–12 weeks old C57Bl/6J mice were purchased from Charles River (Sulzfeld, Germany). B6‐Tg (Rlbp1^GFP^) mice (4–8 weeks old) were originally provided by Prof. Dr. Christian Grimm. Genotyping of Rlbp1^GFP^ mice was performed as previously described (Graca, Hippert, and Pearson 2018). Csf1r^EGFP^ mice were acquired from Jackson Laboratory (Bar Harbor, ME, USA; Strain #005070) and express enhanced green fluorescent protein in resident macrophages and microglia in the retina. All animals were housed in designated animal holding facilities observing a standard 12‐h day/night cycle. Standard rodent chow and water were provided ad libitum.

### 4.2 Retinal Laser‐Injury

Focal injury to the retina was induced as previously described (Conedera, Pousa, et al. 2021). Mice were anesthetized by subcutaneously injecting 45 mg/kg ketamine (Ketalar, 50 mg/mL; Pfizer AG, Zurich, Switzerland) and 0.75 mg/kg medetomidine hydrochloride (Domitor, 1 mg/mL; Orion Pharma AG, Zug, Switzerland). Six lesions were created on each eye using a 532 nm diode laser (Visulas 532 s, Carl Zeiss Meditec AG, Oberkochen, Germany). For the RNAseq analysis, we induced retinal damage by generating up to 50 laser burns per eye, encompassing the visible portion of the fundus. Each burn of 100 μm in diameter was produced with 120 mW of power for 60 ms.

### 4.3 Retinal Dissociation, Sorting, and RNA‐Seq Library Production

As previously described (Conedera, Pousa, et al. 2021; Conedera, Quintela Pousa, et al. 2021), etinas of Csf1r^EGFP^ or Rlbp1^GFP^ mice (*n* = 3 per group) were dissected at Days 1, 3, and 7, and digested with papain (Worthington Biochemical, Freehold, NJ, USA) for 15 min (Feodorova et al. 2015). After dissociation, cell suspension was incubated in HBSS with 0.4% BSA (ThermoFisher Scientific, Basel, Switzerland) and DNase I (200 U/mL; Sigma‐Aldrich, Buchs, Switzerland) and filtered through a 35 μm cell strainer (Costar Corning, Cambridge, MA, USA). To evaluate DNA content, Hoechst 33342 Ready Flow Reagent (ThermoFisher Scientific) was added to the cell suspension. Background fluorescence was determined using cells from Csf1r^EGFP^‐ or Rlbp1^GFP^‐negative littermates.

Before and after injury, we sorted 100 Csf1r^EGFP^‐ or Rlbp1^GFP^‐positive cells/μL using Moflo Astrias (Beckman‐Coulter, Nyon, Switzerland) into Buffer TCL (4 μL; Qiagen, Venlo, Netherlands) containing 1% 2‐mercaptoethanol (Sigma‐Aldrich). We selected proliferating Rlbp1^GFP^ positive cells based on GFP expression, and their DNA content as previously reported (Conedera, Quintela Pousa, et al. 2021). After cell sorting, all samples were processed using the published Smart‐seq2 protocol4 to generate the cDNA libraries (Picelli et al. 2014). Subsequently, the libraries were sequenced on an Illumina HiSeq4000 (Illumina, San Diego, CA, USA) with a depth of approximately 20 million reads per sample. Sequencing data is available upon request.

### 4.4 RNA Sequencing

The raw sequencing reads underwent initial processing, including the removal of adapter sequences, trimming of low‐quality ends, and the exclusion of reads with a phred quality score below 20, achieved through the utilization of Trimmomatic (Version 0.36). Subsequent read alignment was executed utilizing STAR (v2.6.0c). The Ensembl murine genome build GRCm38.p5, augmented with gene annotations retrieved on 2018‐02‐26 from Ensembl (release 91), served as the reference genome for the alignment process. The STAR alignment options were “–outFilterType BySJout –outFilterMatchNmin 30 –outFilterMismatchNmax 10 –outFilterMismatchNoverLmax 0.05 –alignSJDBoverhangMin 1 –alignSJoverhangMin 8 –alignIntronMax 1000000 –alignMatesGapMax 1000000 –outFilterMultimapNmax 50.” Gene expression values were computed with the function featureCounts from the R package Rsubread (v1.26.0). The options for feature counts were: min mapping quality 10 min feature overlap 10 bp—count multi‐mapping reads—count only primary alignments—count reads also if they overlap multiple genes. To identify genes showing differential expression, we employed a count‐based negative binomial model as implemented in the software package DESeq2 (R version: 3.5.0, DESeq2 version: 1.20.0). The assessment of differential expression employed an exact test tailored for over‐dispersed data. Genes displaying altered expression with an adjusted *p* value < 0.05 (Benjamini and Hochberg method) were designated as differentially expressed. Subsequently, heatmaps were constructed for specific gene subsets using the heatmap.2 function from the gplots package (v. 3.0.1) in R v. 3.5.1. These heatmaps visually represented the log2 fold‐changes between the two experimental groups.

### 4.5 Human Specimen and Tissue Processing

Retrospective studies were performed on postmortem eyeballs obtained from donors' eyes after the removal of the cornea for transplantation. Within 24 h postmortem, the whole bulbi without the cornea were fixed with 4% formaldehyde at 4°C overnight, embedded in paraffin and subjected to pre‐established inclusion and exclusion criteria. We included samples from patients of both sexes older than 60‐year‐old. While we acknowledge that many retinal degenerative diseases can have earlier onsets, our focus was on age‐related changes, including but not limited to AMD. We excluded samples from donors suffering from systemic or ocular comorbidities based on the criteria for the corneal donations (e.g., sepsis, meningitis, HIV, lues, hematological neoplasms, all ocular tumors, Creutzfeldt–Jakob disease, rapid progressive dementia or degenerative neurological condition, eye surgery within 6 months or after transplantations, drug abuses). The research was approved by the Ethics Committee of the Canton of Bern, Switzerland (2022‐01842).

Paraffin sections (5 μm) of the peri‐macula was used for histological and immunofluorescence analysis. We selected this region because it retains characteristics relevant to macular degeneration without the confounding factors of the central fovea, which has specialized retinal layers. Additionally, the peri‐macular area is less susceptible to acute swelling, potentially offering a more stable assessment of cellular changes over time (Khan et al. 2016; Li et al. 2018). Overall, Sections were stained with Mayer's hemalum and eosin (H&E; Roth, Karlsruhe, Germany) to evaluate the presence of drusen—extracellular deposits of lipids, proteins, and cellular debris between the RPE and Bruch's membrane—and define the number of nuclei per retina, as previously reported (Gupta et al. 2016; Li et al. 2018; Quinn et al. 2019). Sections were deparaffinized, rehydrated and immersed in a Mayer's hemalum solution for 5 min. Following this, the slides were immersed in an eosin dye (Sigma‐Aldrich). Finally, we rehydrated the sections before mounting them using a mounting medium (Vector Laboratories, Burlingame, CA, USA). Additionally, we stained retinal samples with Picro Sirius red staining specifically designed for histological visualization of collagen fibers and thus tissue fibrosis. For this, paraffin sections underwent deparaffinization and hydration. Subsequently, the nuclei were stained using Weigert's hematoxylin (Roth), for 5 min, followed by rinsing with tap water for 10 min. After, sections were incubated in Direct Red 80 (Sigma‐Aldrich) for 1 h and 30 min allowing it to interact with the collagen fibers. Subsequently, stained samples were washed with acidified water, dehydrated and mounted using the mounting medium for microscopic examination.

High‐throughput and high‐quality brightfield H&E‐ and Sirius Red‐stained images of the human retina were acquired with a NanoZoomer 2.0‐HT slide scanner (Hamamatsu Photonics France, Massy, France). We examined retinas from 30 donors, which were subdivided in two groups (*n* = 15 per group): retinas not showing pathological features of retinal degeneration (control, CTRL) and 15 presenting drusen (> 25 μm), retina atrophy (< average number of cell nuclei) and fibrosis (retina with RD).

### 4.6 Immunofluorescence

Paraffin sections (5 μm) from human and murine eyes were utilized for immunofluorescence. Antigen retrieval was achieved by incubating the sections in either Tris–EDTA (pH 9.0) or citrate buffer (pH 6.0) with 0.05% Tween‐20 for 20 min, then cooling at room temperature for 30 min. Sections were blocked for 1 h in Tris‐buffered saline + 5% goat normal serum + 1% bovine serum albumin (pH 7.6) and incubated with primary antibodies overnight at 4°C. Primary antibodies used in this study were: rabbit anti‐ionized calcium‐binding adapter molecule 1 (Iba1; 1:500; 019‐19741, Wako Pure Chemical Industries Ltd., Osaka, Japan), rabbit anti‐glutamine synthetase (GS; 1:200; ab197024, Abcam, Cambridge, UK), mouse anti‐major histocompatibility complex Class I (MHC I; 1:200; 311402, BioLegend, San Diego, CA, USA), mouse anti‐major histocompatibility complex Class II (MHC II; 1:200; 327002, BioLegend), mouse anti‐inducible nitric oxide synthase (iNOS; 1:200; MA5‐17139, Invitrogen, Carlsbad, CA, USA), and mouse anti‐glial fibrillary acidic protein (GFAP; 1:200; OPA1‐06100, Invitrogen). This step was followed by washing with PBS and 0.05% Triton X‐100 and incubation with the respective secondary antibodies conjugated to 488/594 fluorophores (Alexa Fluor 488 and Alexa Fluor 594, Abcam). Cell nuclei were counterstained using the mounting media Vectashield with DAPI (Vector Laboratories, Newark, CA, USA).

To validate the specificity of MHC I and MHC II staining in paraffin sections, we used both positive and negative controls. The mouse spleen served as a positive control for both markers, given its high expression of MHC molecules, while brain tissue was used as a negative control due to its minimal expression of these markers under noninflammatory conditions. This approach ensures that observed staining is specific to MHC I and MHC II, confirming the reliability of our antibody labeling in these tissues.

Imaging was performed at 40× magnification using a scanning laser microscope (Zeiss LSM710; Carl Zeiss Microscopy, Jena, Germany) on sagittally oriented retinal sections at the level of the laser burn for quantification. The analyzed retinal length included both the damaged area, defined by structural changes in the RPE and photoreceptor layers, and an additional 50 μm beyond the 100 μm damage zone to account for the laser effects on nearby tissue. Positive cells within this defined area were manually counted by a trained observer blinded to the experimental conditions, and quantification was repeated by a second observer to ensure accuracy. Multiple sections from different animals within each experimental group were examined to account for variability, and consistent imaging settings were maintained throughout the study. This methodology was consistently applied across all figures involving similar datasets, ensuring comparability across different experimental conditions and time points.

### 4.7 Cell Lines

The human microglial clone 3 cell line (HMC‐3) was purchased from ATCC (CRL‐3304; Manassas, VA, USA). HMC‐3 cells were cultured in Dulbecco's minimum essential medium (DMEM; Gibco, Scientific, MA, USA) supplemented with GlutaMAX (Gibco), 10% fetal bovine serum (FBS; Gibco) and 1% antibiotic/antimycotic (A/A; Gibco). The spontaneously immortalized MIO‐M1 cell line was provided by Limb et al. (2002). This cell line was cultured in DMEM, to which 10% FBS and 1% A/A was added. Both cell lines were maintained in a humidified incubator at 37°C and 5% CO_2_ and were promptly split as soon as they reached confluency.

### 4.8 Isolation of Human Primary T‐Cells

Primary human T‐cells were isolated from anonymized, donated buffy coats from the Interregional Blood Bank Bern (Project P_406). The separation was performed by centrifugation via a Ficoll‐Paque (Gibco) density gradient. To begin the isolation process, the buffy coat was diluted with Dulbecco's Phosphate‐Buffered Saline (DPBS; Gibco) in a 1:1 ratio in a conical tube (Corning, New York, NY, USA) and mixed gently. Subsequently, the diluted buffy coat was carefully added onto the Ficoll‐Plaque, ensuring that the two phases are not mixed, and centrifuged at 900 g. Following centrifugation, the PBMC layer was transferred into a separate tube containing 10 mL of DPBS, mixed gently, and centrifuged at 250 g. After centrifugation, the supernatant was discarded, and the pellet, containing the cells, was resuspended in Mojo sorting buffer (BioLegend) composed of DPBS supplemented with 1% A/A (Gibco) and 2.5% FBS. Cell count assured that the cell number falls within the range of 1 × 10^7^ to 2 × 10^8^ for using the MojoSort human CD3 T‐cell isolation kit (480022; BioLegend). The procedure was performed as recommended by the manufacturer. In brief, 10 μL of the biotin‐antibody cocktail was added to the filtered cells. The sample was mixed gently and placed on ice for 15 min. Next, 10 μL of streptavidin nanospheres were added to the sample and left on ice for an additional 15 min. Afterward, the sample was placed inside the magnet for 5 min. CD3^+^ T‐cells, which have bound to the streptavidin nanospheres, were then harvested for further analysis. CD3^+^ T‐cells were plated in 24‐well plates (1 × 10^7^ cells per well) and cultured in RPMI medium supplemented with 10% FBS and 1% A/A. To activate CD3^+^ T‐cells, we stimulated them with a human anti‐cluster of differentiation 3 antibody (CD3; 1:200; 300465, BioLegend) and then incubated at 37°C for 72 h.

### 4.9 In Vitro Migration Assay

To determine the migration of T‐cell toward APCs, a transwell assay was performed. To evaluate the effect of activated T‐cells on HMC‐3 and MIO‐M1, a 12‐well tissue culture insert with a pore size of 8.0 μm was utilized (Corning). HMC‐3 cells were detached using trypsin (Gibco), centrifuged, and re‐suspended in serum‐free RPMI medium supplemented with GlutaMAX and 1% A/A. The same procedure was performed for MIO‐M1 cells. HMC‐3 and MIO‐M1 cells were then seeded in the lower chamber (4 × 10^4^ cells per well) and allowed to adhere for 4 h. After the adherence period, T‐cells (1 × 10^6^ per well) were resuspended in serum‐free RPMI medium with 1% A/A and added to the upper chamber. After 3 h of incubation, the migrated T‐cells were collected from the lower chamber. Immunocytochemical staining was conducted on these cells using rabbit anti‐CD4 (1:200; ab133616, Abcam) and mouse anti‐CD8 antibodies (1:100; ab17147, BioLegend) to identify and quantify the migrated T‐cell subsets. This procedure was performed in triplicate.

### 4.10 Activation of Glia Cells

When the HMC‐3 or MIO‐M1 cells reached confluency, they were detached using trypsin at 37°C for 5 min, centrifuged at 300 g for 5 min, and re‐suspended in their media. Subsequently, cells were seeded in 8‐well chambers (Corning, New York, NY, USA) (4 × 10^4^ cells per well), incubated overnight to adhere, and starved in serum‐free media for 6 h. Afterward, cells were treated with lipopolysaccharide (LPS; 2 μg/mL; Sigma‐Aldrich) for 24 h to reach a pro‐inflammatory state. This procedure was performed in triplicate.

### 4.11 Treatment of Glia Cells With Either T‐Cell‐Derived Media or Activated T‐Cells

After activation, T‐cells were centrifuged at 300*g* to separate the supernatant from the cell pellet. The latter, consisting of the activated T‐cells, was gently resuspended in RPMI medium supplemented with 10% FBS and 1% A/A. In the next step, HMC‐3 or MIO‐M1 cells were incubated with the resuspended pellet and the separated supernatants separately. This procedure was performed in triplicate.

### 4.12 Immunocytochemistry

To examine the cellular interactions, immunocytochemistry was performed in an 8‐well chamber slide (Corning) containing HMC‐3 or MIO‐M1 cells in contact with T‐cells and T‐cell‐derived media, respectively. This approach allowed for the visualization and analysis of specific markers, as mouse anti‐iNOS (1:200; MA5‐17139, Invitrogen) and mouse anti‐GFAP (1:200; OPA1‐06100, Invitrogen). To perform this staining, the cells were fixed with 4% paraformaldehyde (Sigma‐Aldrich) at room temperature for 30 min. After DPBS washing, the cells were blocked with 5% normal goat serum (Dako, Glostrup, Denmark) in DPBS supplemented with 0.5% Triton‐X‐100 to minimize nonspecific binding for 30 min. Primary antibodies were added in DPBS, and the cells were incubated at 4°C overnight. The wells were washed three times with DPBS, followed by incubation with the secondary antibodies, goat anti‐rabbit/anti‐mouse Alexa 488/594 (1∶500; Invitrogen) diluted in DPBS at room temperature for 1 h. The nuclei were counterstained with DAPI (1:15,000; Sigma‐Aldrich) at room temperature for 5 min, and the wells were washed again before being mounted with Vectashield mounting medium (Vector Laboratories). The cells were imaged using a confocal microscope (LCI Zeiss LSM 710), and the percentage of positive cells for each marker was calculated using ImageJ software (NIH, Bethesda, MD, USA).

### 4.13 Statistical Analysis

Statistical analysis was performed using GraphPad Prism (version 7.0; GraphPad Software, La Jolla, USA). Intergroup comparisons were based on a nonparametric one‐way analysis of variance (ANOVA) and the Bonferroni multiple comparison post hoc test. All results are expressed as the mean ± standard deviation (SD). The level for statistical significance was set at a *p* value ≤ 0.05.

## Author Contributions

S.I. designed, performed and analyzed cell culture experiments. F.M.C. and D.K. performed immunofluorescence and analyzed RNA‐seq. S.I., F.M.C., J.V.S., and V.E. wrote the manuscript with input from all authors.

## Ethics Statement

Experiments were approved by the local Animal Ethics Committee of the Canton Bern (Switzerland) and conform to the Association for Research in Vision and Ophthalmology Statement for the Use of Animals in Ophthalmic and Vision Research.

## Conflicts of Interest

The authors declare no conflicts of interest.

## Supporting information


**Data S1** Supporting Information.

## Data Availability

All data generated or analyzed during this study are included in this published article and its Supporting Information.
