# Supplementary material for: Glia Modulates Immune Responses in the Retina Through Distinct MHC Pathways
Source: Glia. 2025 Jan 28;73(4):822–39. doi: 10.1002/glia.24656 (PMC11845847; doi:10.1002/glia.24656)
Supplement: Supplementary file 1 — Data S1 Supporting Information. [file GLIA-73-822-s001.docx]

**SUPPLEMENTARY FIGURES**

**Fig. S1: Analysis of MHC proteins by microglia on day 3 after injury.** Shown are representative sections stained for Iba1 (green) and MHC I (red) and monochrome channels. Scale bars equals 100 μm. GCL Ganglion cells layer; INL inner nuclear layer; ONL outer nuclear layer.

**Fig. S2: Gene expression level of molecules playing critical roles in the MHC class II pathway in Csf1r^+^ cells.** RNAseq data quantifying expression of RNAs encoding H2-Eb2, Ciita and Cd74 as fragments per kilobase of transcript per million mapped reads (FPKM).

**Fig. S3: Expression level of Csf genes, interferons and chemokines playing critical roles in the TNF signaling in Csf1r^+^ cells.** RNAseq data quantifying expression of RNAs encoding Csf1r, Ifnar1, Ifnar2, Tnfrsf1a and Tnfrsf1b as fragments per kilobase of transcript per million mapped reads (FPKM). Heatmaps of differentially expressed chemokine-related genes in Csfr1^+^ cells, represented as z-scores.

**Fig. S4: Analysis of MHC proteins by Müller glia on day 7 after injury.** Shown are representative sections stained for GS (green) and MHC I (red) and monochrome channels. Scale bars equals 100 μm. GCL Ganglion cells layer; INL inner nuclear layer; ONL outer nuclear layer.

**Fig. S5: Gene expression level of molecules playing critical roles in the MHC class I pathway in Rlbp1^+^ cells.** RNAseq data quantifying expression of RNAs encoding Psme1, Tapbp and Canx as fragments per kilobase of transcript per million mapped reads (FPKM).

**Fig. S6: Expression level of Csf genes, interferons and molecules playing critical roles in the TNF signaling in Rlbp1^+^ cells.** RNAseq data quantifying expression of RNAs encoding Csf2ra, Ifna7, Ifnar1, Ifnar2, Tnfrsf1a and Tnfrsf1b as fragments per kilobase of transcript per million mapped reads (FPKM). Heatmaps of differentially expressed chemokine-related genes in Rlbp1^+^ cells, represented as z-scores.

**
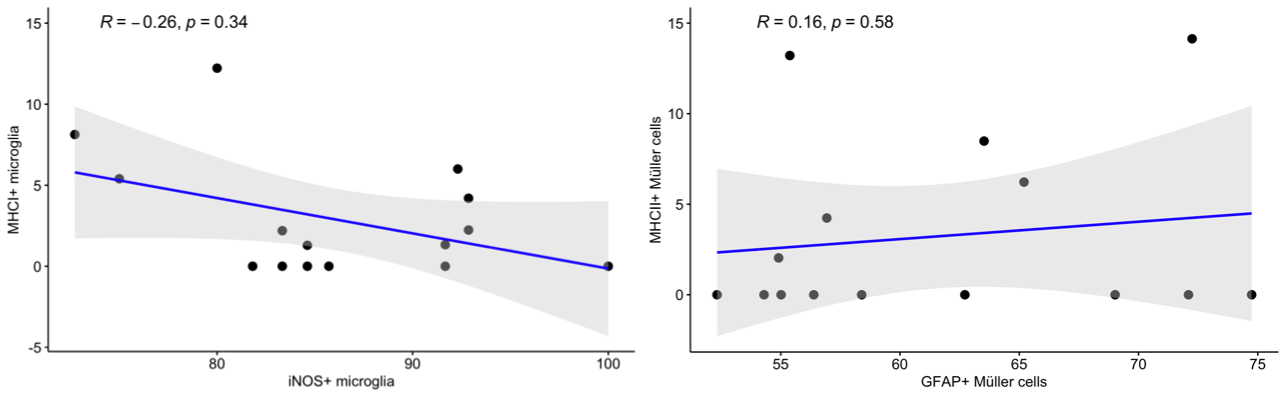
**

**Fig. S7: Relationship between glial reactivity and their expression of MHC molecules.** Spearman correlation between MHC I and iNOS^+^Iba1^+^ cells and MHC II with GFAP^+^GS^+^ cells.
